# Study protocol: older people in retirement villages. A survey and randomised trial of a multi-disciplinary invention designed to avoid adverse outcomes

**DOI:** 10.1186/s12877-020-01640-6

**Published:** 2020-07-17

**Authors:** K. Peri, J. B. Broad, J. Hikaka, M. Boyd, K. Bloomfield, Z. Wu, C. Calvert, A. Tatton, A-M Higgins, D. Bramley, M. J. Connolly

**Affiliations:** 1 School of Nursing, University of Auckland, Private Bag 92 019, Auckland, 1142 New Zealand; 2 Department of Geriatric Medicine, University of Auckland, Level 1, Building 5, Waitemata District Health Board, PO Box 93 503, Auckland, Takapuna 0740 New Zealand; 3 Waitemata District Health Board, PO Box 93 503, Auckland, Takapuna 0740 New Zealand; 4 Auckland District Health Board, Private Bag 92 024, Auckland Mail Centre, Auckland, 1142 New Zealand

**Keywords:** Older people, Retirement villages, Health status

## Abstract

**Background:**

There is increasing interest among older people in moving into retirement villages (RVs), an attractive option for those seeking a supportive community as they age, while still maintaining independence. Currently in New Zealand there is limited knowledge of the medical, service supports, social status and needs of RV residents. The objective of this study is to explore RV facilities and services, the health and functional status of RV residents, prospectively study their healthcare trajectories and to implement a multidisciplinary team intervention to potentially decrease dependency and impact healthcare utilization.

**Methods:**

All RVs located in two large district health boards in Auckland, New Zealand were eligible to participate. This three-year project comprised three phases: The survey phase provided a description of RVs, residents’ characteristics and health and functional status. RV managers completed a survey of size, facilities and recreational and healthcare services provided in the village. Residents were surveyed to establish reasons for entry to the village and underwent a Gerontology Nurse Specialist (GNS) assessment providing details of demographics, social engagement, health and functional status. The cohort study phase examines residents’ healthcare trajectories and adverse outcomes, over three years. The final phase is a randomised controlled trial of a multidisciplinary team intervention aimed to improve health outcomes for more vulnerable residents.

Residents who triggered potential unmet health needs during the assessment in the survey phase were randomised to intervention or usual care groups. Multidisciplinary team meetings included the resident and support person, a geriatrician or gerontology nurse practitioner, GNS, pharmacist and General Practitioner. The primary outcome of the randomised controlled trial will be first acute hospitalization. Secondary outcomes include all acute hospitalizations, long-term care admissions, and all-cause mortality.

**Discussion:**

This paper describes the study protocol of this complex study. The study aims to inform policies and practices around health care services for residents in retirement villages. The results of this trial are expected early 2020 with publication subsequently.

**Trial registration:**

Australia and New Zealand Clinical Trials Registry: ACTRN12616000685415. Registered 25.5.2016.

**Universal Trial Number (UTN):** U111–1173-6083.

## Background

The retirement village (RV) industry provides an attractive housing option for older people who seek a supportive community while still maintaining independence as they age [1]. The term “Retirement Village” in New Zealand (NZ) is also known internationally as a Continuing Care Retirement Community (CCRC), defined as full range lifetime of care from independent to assisted living. The term RV is legally defined in accordance with NZ’s Retirement Villages Act 2003 as “part of any property, building or other premises that contains two or more residential units that provide, or are intended to provide residential accommodation together with services or facilities or both, predominantly for persons in their retirement and their spouses or partners” [2].

There has been a significant growth in RVs in NZ as well as internationally in high resourced countries. From 1988 to 2008, the population of people over 85 years has more than doubled in Auckland, NZ [3]. In spite of this, long-term care (LTC) beds for those requiring 24-h care decreased by almost a third during this same time period, likely due to government policies that promoted community care and “ageing in place” [3–5]. This is likely to have resulted in a trend of increased physical dependency and healthcare complexity for those currently living in RVs [6, 7]. During this same time period, the RV sector saw enormous growth. Recent estimates indicate that there are 380 registered RV complexes in NZ, with 390,000 occupants, a three-fold increase from 1998. Between 2010 and 2016, 40 new RVs opened – a growth rate of 12%. There were almost no RV residents in NZ in the late 1980s, yet by 2017 an estimated 12.6% of people of 75 years and over in NZ lived in RVs [8].

Aside from ‘ageing in place’ and the overall ageing of the population, there are a number of drivers responsible for the increase in demand for RV units (villas/apartments). RV living is growing in popularity with an increasing range of housing and lifestyle ‘products’ to choose from and providing an alternative to traditional lower level residential aged care. Other influences include NZ’s recently strong local housing market and the relative affordability of RV living, and a decline in alternative accommodation options (such as the decline in ageing parents living with their children) [8].

Internationally, RVs have been shown to impact positively on residents’ wellbeing, with higher quality of life compared to those living in family homes or in LTC [9, 10]. NZ research has shown that RV residents are more educated, have more financial resources and better well-being, but have significantly greater functional dependency compared with those in private dwellings [11]. It is clear that only those with substantial financial resources can afford the significant personal investment required initially (NZ$250,000– NZ$1 M+) and the (usually) on-going monthly service fees, [8] resulting in inequity in access.

An increase in the ageing population in NZ is projected over the next decade. The number of older people living within RVs will also likely increase, who as they age will be more prone to increased frailty, greater health needs and declining independence. Indeed, it is likely that RV residents may include a proportion of frailer dependent older people that would have previously resided in LTC facilities [4, 5]. However, little is known in NZ, or elsewhere, about health or functional needs of this potentially vulnerable group of older people, or of what services are or are not provided by RVs to their residents.

In 2012 we completed a small feasibility survey of the functional status, dependency & social circumstances of 110 participants in Auckland RVs [12]. Forty-five percent had been assessed for publicly funded homecare help while 15% received help with personal cares. They had higher morbidity and need (vs. community-dwelling older people), including falls, general health, help with bathing/ dressing, and cognition. These findings suggest that physical function of RV residents lies between those living in private homes and those in LTC.

The basis for the current study was our belief that RV residents may have multiple unmet needs together with declining medical and functional ‘trajectories’ with time, and that a targeted intervention might decrease the risk first acute hospitalisation, number of all acute hospitalisations, LTC admissions and all-cause mortality.

The current study draws on the information gathered from residents themselves, from their healthcare records and at a facility level, to gain an overall better understanding of residents’ health status and of RV service provision. It will inform understanding of characteristics, functional abilities, medical acuity and ‘trajectories’ (actuality and time scales of hospitalisation, move to LTC, mortality) of RV residents. This will, in turn, inform health planning to improve health outcomes in residents. The aims of the current paper are to provide an overall description of the overarching project and detailed methods information.

## Methods/design

### Objectives

We hypothesize RV residents have multiple unmet needs and high healthcare use and that a targeted intervention will decrease the risk of first acute hospitalisation, number of all acute hospitalisations, LTC admissions and all-cause mortality.

The objectives of the study are:

1. To describe general health and functional status of residents;
2. To describe the types of facilities and services that RVs provide;
3. To examine the healthcare service utilization trajectories of RV residents over a three-year period (or longer depending on further funding);
4. To assess in a randomised controlled trial (RCT) the effect of a multidisciplinary team (MDT) intervention on healthcare outcomes in an identified, at-risk subgroup of RV residents.

### Study design

This is a three-year project with data collection commencing in July 2016. This study comprises three phases: Survey phase - a description of RVs and of residents’ characteristics and health and functional status; Cohort study phase - modeling of residents’ health care trajectories up to 3 years (and longer if further funding can be secured); An RCT of a MDT intervention in the most vulnerable cohort of residents, aiming to reduce adverse outcomes. The study was approved by the New Zealand Health and Disability Ethics Committee (Ref 16/CEN/34).

#### Setting

A list of all RVs in the Auckland (ADHB) and Waitematā (WDHB) District Health Boards (that are responsible for providing and/or funding the provision of health services in their defined geographical area) was compiled from a variety of sources (online, previous study data and personal contacts). This list was used as the sampling framework for the recruitment of RVs and we planned that all RVs would be invited to take part regardless of size, ownership or location.

### Recruitment procedures

We planned to approach all RVs in ADHB and WDHB, with random sampling of units/apartments in each village using the villages’ own lists as the sampling frame. Exclusions: refusal of, or inability to consent (Addenbrookes Cognitive Examination Revised ACE-R) [13] under 65, or any resident that our research gerontology nurse specialists (GNSs: AT and CC) or the resident’s RV manager felt may lack capacity to consent. Ethics approval did not require written consent from the facility.

#### Village managers

A GNS allocated to each DHB contacted individual RV managers and gained verbal or email agreement for them to participate in a Village Management Survey. The purpose of this survey is to describe the RVs in the region, in terms of size, facilities and services provided, in order to give a detailed overview of the physical environment: the number of independent-living units or apartments, and ‘serviced apartments’ (units whose residents were able to access from the RV, at additional cost to themselves or to their DHB, services ranging from household help to personal care), recreational facilities accessible to residents, and healthcare services on site.

Where necessary, the Principal Investigator (MJC) contacted the Chief Executive Officers (CEOs) of large RV chains to explain the study and seek permission and access to the RV to improve the participation rate of RV managers and residents.

#### Village residents

Survey phase: We planned to access residents via RV managers, and to contact residents by letter-drop and door-knock. In smaller RVs (*n* ≤ 60 units/apartments), we planned to invite all residents to assess eligibility, whilst in larger RVs we planned to invite residents in a random sample of units (aiming for about 30 residents per RV).

Potential participants were approached using a variety of methods including letter-drop, RV newsletter, noticeboards, and resident meetings and, in larger villages, lists of units from village RV managers for random selection.

RCT resident recruitment: We planned to utilise the InterRAI Community Health Assessment (CHA) (https://www.interrai.org/community-health.html) [14] as a component of the assessment of residents in the Survey phase and to identify residents eligible for the RCT based on these data [15, 16].

As the study evolved and progressed, recruitment issues and other practical problems necessitated changes in the originally designed and funded protocol. These pragmatic changes are detailed in Table 1. Problems and issues specific to subject recruitment will form the subject of a separate publication.

**Table 1:** Summary of planned and actual study design and conduct

	**Planned /expected**	**Implemented /actual**
**Survey Phase:**		
Village sampling frame	Sampling frame would be complete except for new villages opening during the study period and additional units opening in existing villages .	Some villages duplicated in list; and some villages listed were out of area. Villages closed units for renovation, or added units/apartments Facilities were listed that were not retirement villages. The details were updated during the course of the Survey phase.
Village selection	No selection of villages was planned. All villages would be approached, and we expected any decline in participation to be minor.	Slower than anticipated study progress led to recognition that not all villages could be included, A change in policy saw villages being recruited in random order after Dec 2017.
Village recruitment	Gate-keeping role of village managers was recognised, but the extent research team were not given permission to recruit at all was not anticipated.	A high proportion of managers declined participation early in study. This will be detailed in a separate paper.
Unit sampling frame	Within each village we anticipated that a list of all units/apartments (without names of residents) would be provided by village manager, enabling random selection of units, together with permission and security access for door-knocks and letter-drops allowing recruitment to proceed.	Some Managers declined to provide a list of units and also to permit access to doors, making door-knocks or even letter-drops impossible. Instead, these managers permitted study nurses to present an outline of the study at a residents’ meeting, and seek volunteers for participation. This will be detailed in a separate paper as above.
Call-backs	Where at door-knock there was no response, the researchers were to call back up to 5 times, in accordance with Good Practice in Conduct and Reporting of Survey Research [17]. to avoid bias towards those who are frequently at home.	After several months of fieldwork, 10 call-backs was deemed too high a number for the yield provided, and call-backs were reduced to 3.
Resident eligibility criteria	All residents in randomly-selected units would be invited to participate. Further, *every* resident under an LTC contract (i.e. receiving formal publicly-funded LTC services) would be included.	All residents in each randomly-selected unit/apartment were invited. In villages where random selection was not undertaken, all residents were invited. Few residents were in receipt of LTC contracts, and the numbers did not justify continuing this line of enquiry.
Resident sampling	Where more than one resident was in a unit/apartment, and both met resident inclusion criteria, then both were to be invited, to enable assessment of support from co-residence and inter-dependence.	This went to plan.
Informed consent	All residents were eligible to participate regardless of health or occupancy status. For those without capacity to consent but with a legal proxy available, we planned to use the combination of respondent and proxy.	On the basis of the NZ legislation, the ethics committee approval for the study did not permit recruitment of residents who did not have capacity to consent. This will be detailed in a separate paper as above.
Survey instrument(s)	Both feasibility and pilot studies used questionnaires based on our prior research in residential care facilities, based on Booth questionnaire, BRIGHT study and ARCHUS tools.	Mandatory introduction of the interRAI tool nationally was deemed more relevant, appropriate and thorough than our instruments, so the interRAI CHA was added. We selected the interRAI CHA tool (previously unused in NZ) as our main study instrument, and adapted the trialled survey tool as a supplementary survey questionnaire to capture additional items such as reasons for moving into a RV.
Timeline	We planned to begin the Survey phase recruitment on 06June 2016 and complete it by August 2018.	We began Survey phase recruitment on 27 July 2016 and completed in late August 2018.
Sample size and Power calculations	Refer to text.	Refer to text.
Data availability and collection	Survey data were expected to be collected online and immediately available for use in monitoring progress, conducting follow-up and to determine eligibility for the RCT.	Once the decision was made to use an interRAI instrument (where data are captured online and available only occasionally to researchers), it was impossible to use interRAI data directly for determining eligibility for Phase 3. Selected variables were therefore entered into the survey database for this purpose.
**Cohort Follow-up:**		
Timeline	From June 2016 to August 2018	From July 2016 to January 2020
Length of follow-up	Average 1 year	Average 2.5 years
Endpoints of interest	Primary endpoint: First acute hospitalisations, Secondary endpoints: ED presentation, LTC admissions, mortality	Primary endpoint: First acute hospitalisations, Secondary endpoints: number of all acute hospitalisations, LTC admissions, all-cause mortality
Power calculations	Refer to text	Refer to text
**RCT**		
Eligibility criteria	Initially it was planned to recruit those from the Survey phase sample deemed most ‘vulnerable’ (to adverse outcomes) based on selection criteria employed in our ARCHUS study, including the number of medications prescribed.	Once the interRAI tool was chosen, interRAI based criteria were used to select residents, including triggering of 3 or more CAPS, (LTC institutionalisation risk) scores of 3 or more or at the discretion of the GNS. The number of medications reported in the interRAI tool included over-the-counter medications. On investigation, very few residents were being missed if number of medication criteria was dropped, so that inclusion criterion was removed. We retained from the ARCHUS study the validated criterion of the GNS’s impression of vulnerability/medical acuity.
Pre-randomisation checks	All eligible for the trial were to be checked by a study nurse before randomisation to ensure those no longer eligible for the trial were excluded from randomisation.	Survey phase recruitment issues meant that resources to conduct pre-randomisation checks were limited to database checks and short contact with residents in the active arm only, i.e. after randomisation.
Sample size	Refer to text	Refer to text
Timeline	We originally planned to complete the RCT in all villages by 28 Feb 2019	We completed RCT randomisation for in mid-January 2019 and completed the final MDT on 14 March 2019.
Interval between survey and randomisation	The interval between survey (and interRAI assessment) was anticipated as being 3 months, so the assessment would reasonably represent health status at time of randomisation.	An average interval of 9 months between survey and randomisation was achieved. Consequently, some assessments will misrepresent health status at time of randomisation.
Length of follow-up	Planned follow up possibly at 1, 2 & 4 years post-intervention.	Planned follow up 1.5 years post intervention MoH data. The 4th years post-intervention follow-up is conditional on obtaining additional funding
Endpoints of interest	Primary endpoint: First cute hospitalisation Secondary endpoints; All acute hospitalisations, LTC admissions, mortality	Primary endpoint: First acute hospitalisation Secondary endpoints: number of all acute hospitalisation, LTC admissions, mortality Power analyses are based on the primary outcome. We did consider changing the primary outcome to ED presentations, but decided against this as, in NZ, many people who are acutely hospitalised are not admitted via ED but go via GP referral to acute admitting units.
Power calculations	That residents were clustered into villages led to power calculations including an adjustment for a cluster effect.	Clustering was catered for by stratified random sampling, and adjustment for cluster effect was unnecessary.

##### Survey baseline

Recruited residents, after GNSs obtained their written, informed consent, were asked to complete a questionnaire facilitated by the GNSs that provided details of demographic, social engagement, and decision-making paradigms. Items in the resident survey included factors that influenced move to a RV, previous home ownership, home-based support services currently receiving, satisfaction in aspects of RV living, types and extent of social and physical activities currently undertaken, use of dental and other health care services, internet usage, loneliness, spiritual wellbeing and any planned surgery or medical intervention. This questionnaire was informed by our previous feasibility study [12]. Recruitment began in July 2016 and was completed in August 2018.

Where a prior interRAI Home Care (HC) (https://www.interrai.org/home-care.html) or Long Term Care Form (LTCF) (https://www.interrai.org/long-term-care-facilities.html) assessment had been completed within the past 6 months, this assessment was used. The purposes of an interRAI assessment is to accurately determine the characteristics of a person in order to fully describe their needs, ranging from clinical to social support. The outcome measures of the interRAI assessment are known as Clinical Assessment Protocols (CAPS) and are designed to assist the healthcare professional to interpret systematically all the information recorded [14].

We also utilised InterRAI-CHA as a component of assessment in the Survey phase, and for selection for the RCT. “the InterRAI CHA Assessment form is a Minimum Data Set (MDS) screening tool that enables an assessor to review multiple key domains of function, health social support and service use. Particular interRAI CHA items also identify persons who could benefit from further evaluation of specific problems or risks for functional decline. These items, known as triggers, link the interRAI CHA to the problem orientated CAPs. The CAPs provide general guidelines for further assessment and individualised care and services”.

##### Cohort study follow-up

Study outcomes will be to describe trajectories of healthcare utilisation. The primary outcome will be first acute hospitalisation, while secondary outcomes will number of all acute hospitalisations, LTC admissions and all-cause mortality. All are available from routinely collected Ministry of Health (MOH) administrative data, by use of each individual’s unique National Health Identifier (NHI). For any subjects declining entry into the RCT (below), GNSs confirmed their continued permission to obtain these centrally-held data.

##### RCT

Based on a combination of the interRAI assessment and selection criteria validated from our ARCHUS study [15] a sub-sample of resident from the baseline phase (initially estimated at about 28%) ‘at high risk’ of health and functional decline, will be selected for inclusion in the RCT (Table 1). These potentially ‘at risk’ residents, eligible for the RCT, will be identified by triggering three or more interRAI CAPS. There will be no additional exclusion criteria applied. After GNSs obtained written, informed consent, eligible residents will then be randomly allocated to the control or intervention arm, stratified by village. The randomisation list will be generated (using computer-based random number generation) by a statistician not involved in outcome measures. This will ensure that the two groups are balanced regarding each RV. The controls will receive no additional contact, assessments or services. The intervention group will receive a GNS-facilitated intervention plan, similar to our ARCHUS study [15]. In brief, this comprises a comprehensive geriatric assessment by a clinical team consisting of a GNS, a geriatrician and a clinical pharmacist reviewing baseline 1 assessments, residents’ medical records and a further update of health issues prior to the MDT meeting. If an urgent significant health issue is identified, intervention subjects will receive a direct referral from the research team to other relevant services and/or a phone call to the GP. Randomisation and MDTs began in March 2017.

The MDT review will occur in collaboration with the older person & their chosen support person(s), geriatrician or nurse practitioner, and clinical pharmacist. This will culminate in an MDT meeting (usually in the resident’s apartment) lasting approximately 45 min, to which the subjects’ general practitioners (GPs) will be invited. Treatment goals and recommendations will be discussed and developed with the resident and all present, and a summary of the MDT meeting with suggestions and recommendations sent by mail to each participant and their GP.

The effect of the intervention on health outcomes will be assessed by this RCT. Healthcare use (NZ Ministry of Health databases) using the resident’s unique NHI number will be evaluated 1-year pre and 1, 2- & 4-years post-intervention (the latter depending on further research funding being obtained). The Primary Outcome will be first acute hospitalisation. Secondary outcomes will comprise the number of all acute hospitalisations, LTC admissions, and all-cause mortality.

### Data management

The two surveys used online Qualtrics tool (https://www.qualtrics.com). InterRAI assessments are also conducted online, using the same systems as all NZ needs assessments are undertaken. Electronic lists of the unique study identification numbers, date of consent to the study and NHI number will be sent to the interRAI organization and MoH for data extract purposes. Outcome and health service use data will then be provided by interRAI and the MoH by secure link to the researchers, identified by study ID, i.e. without NHI numbers, names or any other identifiers. Data are stored on password-protected shared drives available only to bona fide members of the university research team, and located in locked University offices.

Similarly, health outcomes data for the Survey phase and the RCT will be obtained from MoH from an electronic file supplied by the research team, following an approval process. Data from the two surveys, from interRAI and from MoH will be imported into SAS version 9.4 (SAS Institute Inc., Cary. NC, USA) used for all analyses.

### Power and sample size calculation

Initial power calculation for the RCT was performed prior to commencement of the Survey phase. Following commencement of the Survey phase it became apparent that the subjects were of higher medical acuity and vulnerability than we had anticipated and revised power calculations for the RCT were performed before RCT commencement.

Sample size for the Survey phase follow-up phases was largely governed by the sample needed for the RCT. An estimated 65% of the Survey phase follow-up residents were eligible for the RCT, meaning that 572 residents were needed in the Survey phase. An average of 2.5 years follow-up will be available for the Cohort phase by January 2020. If the rate of acute hospitalisation (primary outcome) is 55% per year we will have at least 80% power to show a 20% difference in the hazard ratio for any two categories.

For the RCT, we estimated 372 residents met the “high-risk” criteria, will be randomised and followed for 1 year after randomisation. The rate of acute hospitalisation in LTC residents is 67% per year (from previous work). We conservatively hypothesised the acute hospitalisation rate in the “high risk” RCT RV residents would be 70% per person-year. We would expect 80% power to show a 20% reduction in our primary outcome (acute hospitalisation). If the effect is stronger (e.g. 25% or 30% - as found in our ARCHUS [16] and ARCHIP [18] studies or longer follow-up, power will increase, though based on our earlier work (ARCHIP), the duration of any intervention effect may be under 1.4 years.

### Statistical analysis

In the Survey phase, initial descriptive analyses will be conducted. Then, in both the Survey and Cohort phases, multivariate regression models (including logistic regression, log-binomial regression, linear regression and Cox regression) will be used to explore the influence factors on health outcomes adjusting for potential confounders (demographics, lifestyle, medical history, clinical characteristics, village characteristics etc.) and time to event when possible.

For the primary and main secondary outcomes in the RCT, analyses will be conducted on an intention-to-treat basis. Cox proportional regression models, with robust sandwich variance estimates, will be used to compare the time to health outcomes between the intervention and usual care groups. Hazard ratios (HRs) and 95% confidence intervals (CIs) will be calculated. Sub-group analyses will evaluate treatment effect for the main endpoint for different sub-groups, e.g. subgroup analyses for each endpoint by sex, large/small village, clinic/nurse/not, sampled/volunteers. Analyses will be conducted by a research statistician not involved in the clinical aspects of the study.

### Study status

Recruitment to the Survey phase ended in August 2018**.** RCT randomisation was completed in mid-January 2019 and all MDTs were completed by 14 March 2019. This will give us, by end of January 2020, a median (range) 29 (18–42) months of Cohort follow-up and 18 (12–33) months RCT follow-up. Trial results will be the subject of further publications and communicated to residents by regular newsletters.

## Discussion

The aims of the Older People in Retirement Villages project are to describe RVs within the study region and the health and functional status of residents (Survey phase); to examine these over a two-to-four-year period (Cohort follow-up); and to assess the effect of a MDT intervention in an identified at risk group of residents (RCT) on the healthcare outcomes of first acute hospitalisation, all acute hospitalisations, LTC admission and all-cause mortality. It is the largest cross-sectional study of RVs in NZ and has enabled a longitudinal cohort study of healthcare utilisation and a randomised controlled trial of comprehensive gerontology intervention. Its potential weakness relates to difficulties in recruitment of both retirement villages and of village residents, potentially affecting representativeness of recruited subjects and summarized in Table 1. These issues will form the basis of a separate paper.

The findings from this project will represent the first comprehensive portrayal of older RV residents NZ and an overview of RV settings. The results from this study will help inform policy development and health care provision strategies, as it will provide a clearer picture of unmet need and the effect of a targeted intervention in this population. This trial will be of interest to service users (residents and potential residents) illustrating how social and physical environmental settings are characterized, and possibly how the older population are ageing in RVs compared to other conglomerate housing settings.

In addition, social connection and participation are considered an aspect of RV living that flourishes [19]. A recent NZ study involving 163 RV residents in two RVs found that a “dignified” environment improved mental wellbeing, provided an environment that built relationships and reduced loneliness and isolation [11]. However, some international studies have shown that family contact reduces for village residents compared to community dwelling older people. Given the large and increasing numbers of older New Zealanders living in RVs, it is surprising and potentially concerning that we know so little about them. The current study will further explore these areas and its results could have wider ranging implications for future service planning for RV residents, including but not restricted to those who develop complex physical or cognitive needs. RVs are not unique to NZ and our findings will inform planning and provision internationally.

Our previous work in the LTC sector indicates that targeting of vulnerable, co-morbid older people and offering GNS-led, complex intervention reduces unnecessary hospitalisations for many conditions by over 20% (vs. usual care) [16, 18]. It is feasible that such interventions (as in the current study’s RCT) will similarly benefit RV residents.

We anticipate that our findings and experiences will also provide insights into the opportunities and challenges of collaboration with RVs and provide recommendations to future research partnerships.

## Data Availability

Not applicable for this paper because no data has been generated. Participants are and will be updated regarding study results by newsletter.

## Abbreviations

RVs: Retirement villages
NZ: New Zealand
MDT: Multidisciplinary team
GNS: Gerontology nurse specialist
RCT: Randomised controlled trial
CCRC: Continuing care retirement community
LTC: Long-term care
ED: Emergency department
ACE-R: Addenbrookes cognitive examination revised
CHA: Community health assessment
HC: Home care
LTCF: Long term care form
CAPS: Clinical assessment protocols
MDS: Minimum data set
CAPs: Clinical assessment protocols
MOH: Ministry of health
NHI: National health Identifier
GPs: General practitioners
HRs: Hazard ratios
CIs: Confidence intervals
